# Machine learning and computational approaches to model therapeutic response and resistance in diffuse midline glioma

**DOI:** 10.1007/s11060-026-05817-8

**Published:** 2026-09-29

**Authors:** Océane A. Dubois, Olive E. L. Loughnan, Yuanhao Jiang, Bella Petraello, Alicia M. Douglas, Tabitha McLachlan, Izac J. Findlay, Tuan Vo, Clara Savary, Matthew D. Dun

**Affiliations:** 1 Cancer Signalling Research Group, School of Biomedical Sciences & Pharmacy, College of Health, Medicine and Wellbeing, University of Newcastle, Callaghan, NSW Australia; 2 Precision Medicine and Health Program, Hunter Medical Research Institute, New Lambton Heights, NSW Australia; 3 Paediatrics, Mark Hughes Foundation Centre for Brain Cancer Research, College of Health, Medicine and Wellbeing, University of Newcastle, Callaghan, NSW Australia; 4 Institut des Sciences Exactes et Appliquées, Université de la Nouvelle-Calédonie, Nouméa, New Caledonia New Caledonia

**Keywords:** Diffuse midline glioma, Therapeutic resistance, Machine learning, Bioinformatics, Single-cell RNA sequencing, Spatial transcriptomics

## Abstract

**Purpose:**

Diffuse midline glioma (DMG) remains one of the most lethal cancers affecting children, adolescents and young adults and is near-universally resistant to treatment. Histone H3-alterations establish a profoundly dysregulated epigenetic landscape that promotes extensive intratumoral heterogeneity and cellular plasticity, key drivers of therapeutic resistance and treatment failure. Although single-cell RNA sequencing and spatial transcriptomics have transformed the study of tumor evolution, the mechanisms underpinning treatment resistance in DMG remain poorly understood. This Review examines how computational and machine learning-based approaches can be leveraged to study tumor adaptation under therapeutic pressure.

**Methods:**

We provide an overview of computational frameworks developed for the analysis of single-cell and spatial transcriptomic datasets to model four major axes of tumor evolution: *i*) compositional shifts, *ii*) functional state remodeling, *iii*) tumor plasticity, and *iv*) intercellular communication; while highlighting how these approaches provided novel insights into DMG biology and the mechanisms underlying treatment adaptation.

**Results:**

Computational and machine learning-based frameworks provide powerful tools for modeling the spatiotemporal dynamics of tumor evolution in high-dimensional transcriptomic data. Across the four dimensions examined, these approaches identify resistant cellular populations, characterize adaptive transcriptional programs, reconstitute cell-state transitions, and map tumor-microenvironment interactions, revealing mechanisms of therapeutic resistance and treatment adaptation.

**Conclusion:**

Computational approaches developed to model tumor evolution under therapeutic pressure using single-cell and spatial transcriptomic data are rapidly advancing. As increasingly large and multimodal DMG datasets become available, the application of these approaches may help uncover resistance mechanisms, therapeutic vulnerabilities, and biomarkers that inform more precise and patient-specific treatment strategies.

**Supplementary Information:**

The online version contains supplementary material available at https://doi.org/10.1007/s11060-026-05817-8.

## Introduction

Diffuse midline glioma (DMG), including diffuse intrinsic pontine glioma (DIPG), H3K27-altered, is a lethal central nervous system (CNS) tumor arising within the midline structures, and occurs predominantly in children, adolescents, and young adults [1]. The disease is defined by alterations in histone H3 lysine 27, mostly through H3K27M mutations, but also through *EZHIP* overexpression [2]. These molecular events converge on dysregulation of polycomb repressive complex 2 (PRC2)-mediated chromatin regulation, driving the hallmark global reduction in H3K27 trimethylation (H3K27me3) and profound epigenetic and transcriptional reprogramming, including aberrant reactivation of developmental transcriptional programs normally silenced following lineage specification [3]. Single-cell transcriptomic and epigenomic studies show that DMG arises within a developmentally restricted glial progenitor context and recapitulates an oligodendroglial differentiation hierarchy, dominated by proliferative oligodendrocyte precursor cell (OPC)-like states [4, 5]. Extensive cellular heterogeneity and phenotypic plasticity remain major barriers to treatment, as therapy can select for or induce resistant cellular states that remain poorly characterized in DMG.

DMG accounts for 10–20% of pediatric CNS tumors and is the leading cause of cancer-related death in children, with a median overall survival of only 11 months, and fewer than 10% of patients surviving beyond two years [6, 7]. Despite decades of research, radiotherapy (RT) remains the sole standard-of-care treatment, providing only transient clinical benefit [8]. In August 2025, the United States Food and Drug Administration (FDA) granted accelerated approval to dordaviprone (ONC201; MODEYSO™) for previously treated adult and pediatric patients following disease progression aged one year and older with H3K27M-mutant DMG [9]. Preclinical evidence supporting combination with the PI3K/mTOR inhibitor paxalisib also provided a rationale for the adaptive Phase II PNOC022 trial [10]. However, the variable and often ephemeral responses observed among patients raise a central unanswered question: which molecular programs drive DMG adaptation, evolution, and resistance under therapeutic pressure? In a disease with few effective treatment options, understanding and anticipating these adaptive and evolutionary trajectories will be essential to overcome resistance and to develop more durable treatment strategies.

Cancer can be viewed as a dynamic system that changes over time. By analyzing time-series transcriptomics data we can map how tumor cells move between different molecular states. Treatment resistance emerges when cells transition into stable resistant cellular states, known as attractor states. Graph-based approaches can reconstruct these evolutionary trajectories and identify the attractor states associated with resistance, providing a powerful framework for discovering biomarkers and novel therapeutic vulnerabilities [11]. Within a tumor, transcriptionally distinct malignant states exhibit different therapeutic sensitivities, allowing relatively resistant populations to persist and expand following treatment [5, 12]. Therapeutic exposure may also reshape tumor composition by selecting pre-existing cellular states or subpopulations while simultaneously inducing adaptive transcriptional and chromatin-state transitions [13, 14]. Phenotypic plasticity has similarly emerged as a central driver of resistance in high-grade gliomas (HGG), allowing malignant cells to dynamically transition between cellular states, reactivate developmental programs, and adopt alternative differentiation states [5, 15]. Importantly, signals from the tumor microenvironment (TME) promote adaptive responses through dynamic cell-cell communication networks. These processes, including selection, functional reprogramming, plasticity and microenvironmental rewiring, provide a biological framework for understanding therapeutic resistance in DMG.

The transcriptome provides a functional readout of cellular identity and underlying signaling programs, offering insight into tumor heterogeneity and adaptation to therapy [16]. Single-cell RNA sequencing (scRNA-seq) resolves transcriptionally distinct cell populations but loses spatial context and may introduce dissociation-related biases, whereas spatial transcriptomics preserves tissue architecture, often at the cost of transcriptional depth [17]. Interpretation of these complementary, high-dimensional datasets requires sophisticated computational approaches capable of modeling tumor dynamics and therapeutic response [18, 19].

Machine learning (ML), a subset of artificial intelligence (AI), encompasses computational approaches that learn patterns and relationships from data to support classification, prediction, and biological discovery [18, 19]. Approaches can be supervised, using labeled examples, or unsupervised, identifying patterns without prior labels [20]. In transcriptomics, ML and deep learning (DL) approaches are increasingly used for dimensionality reduction, cellular classification, representation learning, network inference and perturbation-response modeling [18]. DL, a subset of ML, employs multi-layer neural network architectures such as autoencoders and graph neural networks (GNNs) to learn complex nonlinear representations of cellular states and spatial relationships [21]. Unlike conventional ML approaches, which typically represent each cell or gene as independent feature vectors, GNNs and related architectures such as graph convolutional networks (GCNs) and graph attention networks (GATs) directly incorporate relational structure between cells or genes into the model. By leveraging cell-cell or gene-gene topologies, network analysis-based approaches are capable of identifying highly connected nodes or coherent subnetworks, that can constitute plasticity biomarkers or therapeutic targets [22]. These approaches complement, rather than replace, established statistical frameworks. Optimal transport (OT) provides a mathematical framework for coupling probability distributions of cells across conditions, timepoints, or spatial locations and is increasingly applied to infer cell-state transitions and spatiotemporal dynamics [23, 24].

**Fig. 1:**
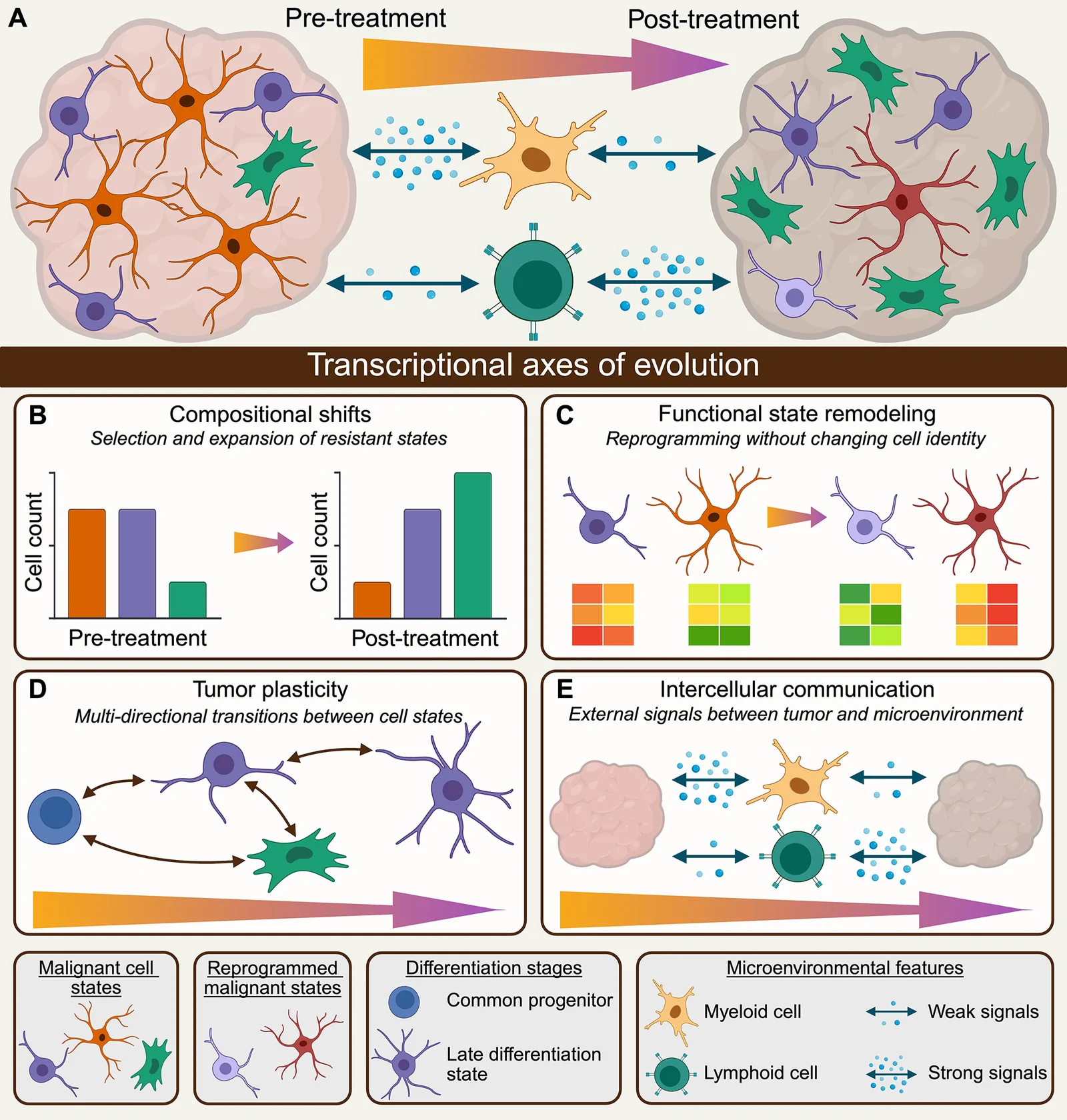
Four axes of tumor adaptation captured by single-cell and spatial transcriptomic profiling. (**A**) Tumors undergo profound reprogramming in response to therapeutic exposure through selection of pre-existing cellular states, induction of adaptive transcriptional programs, dynamic transitions between cellular identities, and rewiring of intercellular networks. (**B**) Compositional shifts reflect the selection, or expansion of resistant cellular populations and states within the tumor post-treatment. (**C**) Functional state remodeling captures transcriptional reprogramming that occurs without a change in cellular identity. (**D**) Tumor plasticity denotes multi-directional transitions between differentiation states or across distinct lineages. (**E**) Intercellular communication changes reflect modifications in the strength and nature of signaling between tumor cells and microenvironmental populations

In this Review, we provide an overview of computational frameworks developed to model tumor adaptation under therapeutic pressure using single-cell and spatially resolved transcriptomic data, with a particular emphasis on their relevance to DMG. Transcriptomic features of tumor evolution are grouped into four axes: *i*) compositional shifts in cellular populations, determining which cell populations or states resist under treatment; *ii*) functional state remodeling, capturing transcriptional programs expressed in persistent cells; *iii*) malignant cell plasticity, modeling transitions between cellular states; and *iv*) intercellular communication networks, inferring signaling interactions that support treatment resistance (Fig. 1). For each axis, we examine established computational tools and the biological insights they provide, prior to discussing their potential to inform treatment development or patient-specific therapeutic strategies.

## Compositional shifts in cellular populations

DMG comprises diverse malignant cell states, predominantly OPC-like, alongside oligodendrocyte-like (OC-like), astrocyte-like (AC-like), and mesenchymal-like (MES-like) populations [5]. The relative abundance and spatial organization of these states vary across patients, anatomical locations and developmental context, further reshaped by treatment [5]. Characterizing tumor compositional shifts remains challenging due to both biological and technical factors. Cellular composition can be influenced by sample heterogeneity, tissue collection, storage and processing, sequencing platforms, and lack of biological replicates [5, 17, 25, 26]. Since abundance is measured as a relative proportion, depletion of one population ultimately results in the apparent enrichment of others [25, 26]. Moreover, conventional clustering approaches may overlook treatment-induced changes by forcing cells into discrete groups, even though cellular states often exist along a continuum [4, 5, 25]. To address these limitations, statistical and ML frameworks have therefore been developed to model differential abundance while accounting for sample-level variability and compositional effects [25, 27]. In various methods, such as Milo, cellular states are represented as “neighborhoods” within a k-nearest neighbors graph, enabling the detection of responsive intermediate states (Table 1, Supplementary Table S1) [25]. In oncology, this strategy has revealed progenitor-like and proliferative tumor populations associated with disease-specific subgroups [28]. More recently, ML-based approaches, including Cellograph, have been developed to identify treatment-responsive and -resistant cellular states across complex biological conditions [29]. Together, these approaches provide a framework for determining which tumor populations are likely to persist under therapeutic pressure.

Importantly, compositional changes are often not uniformly distributed throughout a tumor, but are instead spatially organized in regions defined by specific cellular composition, structures, and cell-cell interactions, commonly referred to as niches [27]. In DMG, proliferating OPC-like and OC-like cells preferentially cluster together within mitotic niches surrounded by more differentiated, non-proliferating AC-like cells. MES-like tumor cells colocalize with vascular cells [27] and *SPP1*+/*GPNMB*+ myeloid cells, constituting an immunosuppressive niche [30]. Therefore, spatially informed approaches capable of identifying niches that are differently enriched across biological conditions are highly relevant in DMG. Emerging methods such as NicheCompass can help elucidate how therapies remodel tumor architecture by identifying novel niches and niche-specific interactions [31].

**Table 1:** Representative computational tools for characterizing tumor evolution

Tool name	Category	Omics type	Description	Method*
Milo (Dann et al., 2022) [25]	Compositional shifts	scRNA-seq	Detects differentially abundant cell states without relying on discrete clustering	Statistical: overlapping k-NN graph neighborhoods; negative-binomial GLM on per-sample neighborhood counts; graph-adapted weighted FDR (“spatial FDR”) that accounts for neighborhood overlap
Cellograph (Shahir et al., 2024) [29]	Compositional shifts	scRNA-seq	Quantifies how “prototypical” single cells are of an experimental condition and visualizes condition-driven variability	DL: semi-supervised GNN (GCN classifier) trained on a cell-cell similarity graph; learns a latent embedding and gene-weight matrix
NicheCompass (Birk et al., 2025) [31]	Compositional shifts	ST (multi-platform)	Identifies and characterizes cellular niches by modeling CCC, producing interpretable embeddings of signaling events across single or multi-sample datasets	DL: constructs a spatial neighborhood graph from cell/spot coordinates; GNN encoder with multimodal conditional variational GAE learning niche embeddings from features plus covariates
RECODR (Jassim et al., 2025) [32]	Functional state remodeling	scRNA-seq	Detects shifts in gene co-expression “context” (rather than expression level) associated with cancer treatment resistance	ML: builds single-cell gene co-expression graphs per condition and applies graph-embedding methods to quantify contextual drift of genes across treatment states
TransMarker (Keikha et al., 2025) [33]	Functional state remodeling	scRNA-seq	Identifies dynamic network biomarkers (genes with shifting regulatory roles) across cancer progression stages from time-course single-cell data	DL: per-state multilayer gene graphs; GAT embeddings; Gromov-Wasserstein OT to score per-gene structural shift; DNI-based biomarker ranking; DNN classifier to validate biomarkers
CancerTrace (Atitey et al., 2025) [34]	Functional state remodeling	scRNA-seq	Recovers dynamic, patient-specific gene regulatory drivers and their upstream modulators across cancer stages from scRNA-seq	Statistical: variational Bayesian model with transfer entropy + sparse conditional structure to map modulator-driver influence across stages
C-SIDE (Cable et al., 2022) [35]	Functional state remodeling	ST (Slide-seq V2, MERFISH & Visium)	Detects cell-type-specific DEG along spatial/biological covariates in ST with cell-type mixtures (labelled previously using tools such as RCTD)	Statistical: models observed spot expression as an additive mixture of log-linear, cell-type-specific expression functions; hypothesis testing framework
SPATA2 (Kueckelhaus et al., 2024) [36]	Functional state remodeling	ST (Visium)	Toolbox for ST analysis enabling user-defined spatial trajectories, allowing gene/gene-set expression to be screened along a spatial axis (e.g., tumor core to infiltrated margin)	Statistical: manually or semi-automatically drawn spatial trajectories with expression values binned/interpolated along trajectory distance; screening compares observed expression gradients against predefined ideal patterns (e.g., linear, logarithmic, gradient descending/ascending) using regression-based fit statistics
Monocle 2 (Qiu et al., 2017) [37]	Tumor plasticity	scRNA-seq	Reconstructs branched/complex differentiation trajectories in an unsupervised way	ML: reversed graph embedding to jointly learn a low-dimensional manifold and a principal tree structure
Slingshot (Street et al., 2018) [38]	Tumor plasticity	scRNA-seq	Infers lineage structure and pseudotime across branching cell trajectories	Statistical: cluster-based minimum spanning tree defines lineage topology, then fits simultaneous principal curves per lineage for pseudotime
PRESCIENT (Yeo & Saksena et al., 2021) [39]	Tumor plasticity	scRNA-seq	Models stochastic cell differentiation dynamics and predicts future cell states from time-series data	DL: models cell state changes as an SDE whose drift is the negative gradient of a neural-network potential function; trained by simulating cell populations forward from the first timepoint and minimizing an entropy-regularized OT (Sinkhorn) divergence to the observed cells at each later timepoint, with pre-specified proliferation rates weighting the cells
GRNvelo (Zhang et al., 2026) [40]	Tumor plasticity	scRNA-seq	Reconstructs multiscale cell-fate dynamics by coupling GRN-driven single-cell velocity (derived from GRN ODEs rather than splicing) with nonlocal, growth-mediated population dynamics in an ODE-PDE model	DL: two-phase PINN optimization. TC-PINN fits Hill-function GRN ODEs under a latent-time ordering constraint to infer GRN velocity and latent time, and MP-PINN refines the velocity using cell density, growth and PDE constraints
CytoTRACE2 (Kang et al., 2025) [41]	Tumor plasticity	scRNA-seq	Predicts absolute developmental potential (potency) and continuous differentiation states of cells	DL: Interpretable neural network (GSBN modules) that decodes potency-associated gene programs to predict discrete potency categories and a continuous potency score
STIFT (Qi et al., 2025) [23]	Tumor plasticity	ST (Stereo-seq, Visium & MERFISH)	Integrates spatiotemporal transcriptomics across timepoints, removing batch effects while preserving developmental trajectories	DL: three-component framework combining developmental spatiotemporal OT, spatiotemporal graph construction, and a GATE informed by temporal triplet learning
Moscot (Klein et al., 2025) [24]	Tumor plasticity	Multimodal; ST (MERFISH & Stereo-seq), CITE-seq, scRNA-seq & scATAC-seq	General-purpose, scalable OT framework for mapping cells across time, space, and modalities in large single-cell atlases	OT: entropic, unbalanced, and fused Gromov-Wasserstein OT formulations covering temporal, spatial, spatiotemporal, and cross-modality mapping problems; low-rank solvers for scalability
STORIES (Huizing et al., 2026) [42]	Tumor plasticity	ST (Stereo-seq)	Learns cell-fate/differentiation landscapes from time-series ST	DL: Wasserstein gradient-flow learning; a neural-network differentiation potential trained using (spatially informed), fused Gromov-Wasserstein OT as the loss
SOCS (Bryan et al., 2026) [43]	Tumor plasticity	ST (Stereo-seq, Slide-seq & MERFISH)	Infers biologically coherent developmental trajectories in time-series ST by preserving contiguous tissue structures	OT: structurally constrained unbalanced Gromov-Wasserstein OT combining gene-expression, geometric, and structural-contiguity penalty terms
CellChat v2 (Jin et al., 2024) [44, 45]	Intercellular communication	Multimodal; ST & scRNA-seq	Infers, quantifies, and visualizes CCC networks from transcriptomic data	Statistical: mass-action-based model (Hill function) using curated multi-subunit L-R-cofactor database; permutation testing for significance; network/pattern-recognition analysis
CellPhoneDB v5 (Troulé et al., 2025) [46]	Intercellular communication	Multimodal; ST, scRNA-seq & scATAC-seq	Infers CCC from single-cell data using a curated L-R repository	Statistical: permutation testing on mean L-R expression per cell-type pair; incorporates spatial microenvironment and TF-activity data to prioritize interactions
CELLetter (Wu et al., 2025) [47]	Intercellular communication	scRNA-seq	Identifies context-specific L-R interactions beyond static, predefined databases and models downstream signaling	DL: protein language model (ProstT5) embeddings, dual-stream feature-extraction network with gated fusion, combined with L-R co-expression and inferred TF activity
Tensor-cell2cell (Armingol et al., 2022) [48]	Intercellular communication	scRNA-seq	Finds recurring, context-dependent (e.g., across samples/time) patterns of CCC from multiple L-R scores	ML: non-negative tensor component analysis of 4D tensors that contain a communication score for each sender/receiver cell type, for multiple L-R pairs, across multiple conditions/contexts
COMMOT (Cang et al., 2023) [49]	Intercellular communication	ST (MERFISH, STARmap, seqFISH, Slide-seq & Visium)	Infers spatial CCC accounting for L-R competition and spatial distance constraints	OT: collective OT formulation jointly resolving multiple competing L-R species and their spatial transport costs
CellNEST (Zohora et al., 2025) [50]	Intercellular communication	ST (Visium, Xenium & MERFISH)	Detects CCC, including multi-hop “relay” networks of sequential L-R signaling, in ST	DL: GAT encoder trained with DGI contrastive self-supervised learning on a spatial knowledge graph
DualCellChat (Hu et al., 2026) [51]	Intercellular communication	ST (seqFISH, MERFISH, HDST & Stereo-seq)	Reconstructs a complete, directed cell-cell interaction network from incomplete ST data	DL: directed GAE, using graph convolution networks, that model directional interactions

*Methods based primarily on statistical inference, mathematical optimization, or probabilistic modeling are classified as Statistical. OT-based methods are mathematical frameworks at the interface between Statistical and ML-based approaches, as they compare probability distributions directly, without a statistical inference procedure (e.g., no hypothesis test, no likelihood) nor a trained predictive model, and are therefore classified in a separate “OT” category [52]. Methods are classified as ML when they explicitly incorporate an ML component involving learned representations, predictive modeling, or algorithmic training, while methods relying on neural network architectures are classified as DL, even if they use OT [18]. Abbreviations: CCC = cell-cell communication; CITE-seq = cellular indexing of transcriptomes and epitopes sequencing; DEG = differentially expressed genes; DGI = deep graph infomax; DL = deep learning; DNI = dynamic network index; DNN = deep neural network; FDR = false discovery rate; GAE = graph autoencoder; GAT = graph attention network; GATE = graph attention autoencoder; GCN = graph convolutional network; GLM = generalized linear model; GNN = graph neural network; GRN = gene regulatory network; GSBN = gene set binary network; HDST = high-definition spatial transcriptomics; k-NN = k-nearest neighbors; L-R = ligand-receptor; MERFISH = multiplexed error-robust fluorescence in situ hybridization; ML = machine learning; MP-PINN = multi-physics physics-informed neural network; OT = optimal transport; ODE = ordinary differential equation; PDE = partial differential equation; PINN = physics-informed neural network; RCTD = robust cell type decomposition; seqFISH = sequential fluorescence in situ hybridization; scATAC-seq = single-cell assay for transposase-accessible chromatin sequencing; scRNA-seq = single-cell RNA sequencing; SDE = stochastic differential equation; ST = spatial transcriptomics; STARmap = spatially resolved transcript amplicon readout mapping; TC-PINN = temporally consistent physics-informed neural network; TF = transcription factor

## Functional state remodeling

Functional state remodeling describes changes in transcriptional programs and regulatory networks within a cell population without altering cellular identity [5, 14, 15]. This distinction is important as a cellular population may persist post-treatment while experiencing profound reprogramming in response to therapeutic pressure [53]. Additionally, such changes may be restricted to spatially distinct niches [27]. Capturing this remodeling may reveal mechanisms of resistance that would remain undetected by compositional analysis alone.

The H3K27M mutation drives extensive metabolic reprogramming in DMG, creating dependencies on specific metabolic programs that constitute therapeutic vulnerabilities [54]. However, the adaptive changes in these pathways caused by treatment exposure can also confer resistance [12]. For example, inhibition of oxidative phosphorylation using phenformin extends survival in preclinical models, although tumors ultimately adapt through activation of alternative metabolic pathways including cholesterol synthesis [55]. Similarly, DMG-H3K27M cells exhibit a strong dependence on purine metabolism [56]. Following RT, DMGs adapt by upregulating the purine salvage pathway. Targeting hypoxanthine-guanine phosphoribosyltransferase (HGPRT) in combination with RT results in durable responses in murine models. However, current studies often provide static snapshots of treatment response, limiting insight into how transcriptional and regulatory programs are dynamically remodeled during tumor adaptation.

To address this challenge, computational approaches have been developed to identify how changes in gene programs and regulatory networks are remodeled during treatment and disease progression (Table 1, Supplementary Table S1). Methods including RECODR can detect shifts in pathway activity, uncover treatment-induced dependencies, identify transcription factors and regulatory circuits that drive adaptation to therapy [32]. Tools such as TransMarker and CancerTrace can reconstruct dynamic regulatory networks to identify candidate genes and pathways associated with these transcriptional changes [33, 34]. Together, these approaches reveal molecular drivers and regulatory changes accompanying tumor evolution.

To characterize TMEs, spatial transcriptomics shows how local niches shape DMG transcriptional programs. Since spatial measurements capture mixtures of cell types, methods such as C-SIDE address cellular heterogeneity by identifying cell-type-specific expression patterns while accounting for cellular composition [35]. Complementing this approach, tools including SPATA2 provide spatial methodologies to infer gene expression gradients in a tissue without establishing precise boundaries, enabling the assessment of gradual changes from a region of interest, such as the tumor core through to the infiltrative margins [36]. These approaches therefore extend functional state analysis by correlating sensitive and resistant regions with transcriptional programs that promote response or tumor adaptation.

## Tumor plasticity

Plasticity represents a hallmark of glial malignancies, enabling malignant cells to transition between transcriptional states in response to developmental, environmental or therapeutic cues [4, 15]. In DMG, H3K27 alterations are associated with metabolic rewiring that increases levels of α-ketoglutarate, an essential cofactor for histone demethylases, thereby reinforcing H3K27 hypomethylation [54]. Dordaviprone disrupts mitochondrial homeostasis and alters α-ketoglutarate availability, partially restoring H3K27 methylation, demonstrating that epigenetic states are therapeutically modifiable [57]. Therapy-induced epigenetic remodeling may therefore contribute to dynamic transitions between malignant cell states, and hence, plasticity-focused approaches, including trajectory inference and pseudotime analyses, RNA velocity and cell fate prediction, offer means to track how DMG cells transition between malignant states under therapeutic pressure [10].

Trajectory inference methods, such as Monocle and Slingshot, order cells along a transcriptional continuum that represents a more dynamic biological process (Table 1, Supplementary Table S1) [37, 38]. In DMG, these approaches have identified multiple OPC-like malignant cell states that differ in their degree of differentiation, with more immature OPC-like-2 and OPC-like-3 populations and a more lineage committed OPC-like-1 population. The relative abundance of these states varies by anatomical location [5]. In DIPG models, these analyses revealed a resistance-associated trajectory, suggesting a model of cell fate reprogramming and identifying resilient MES-like and mitotic populations post-triple therapy [58]. However, pseudotime estimates from static expression profiles provide an ambiguous temporal ordering, and cannot directly assess transition directionality, differentiation pace, or more complex patterns of cell-state change [38, 42, 43].

Therefore, RNA velocity-based approaches leveraging deep generative models were developed, with the potential of reconstructing trajectory direction based on spliced-to-unspliced mRNA ratios. To model complex perturbations at different timepoints, PRESCIENT models potential future cellular-state distributions following perturbation [39]. More recently, GRNvelo integrates gene-regulatory-network dynamics with velocity-like information to model cell-fate responses [40]. CytoTRACE2 provides an interpretable DL framework that is powerful enough to predict the absolute differentiation potential of a cell, spanning from pluripotent to terminally differentiated populations [41]. Expanding insights into cell potency could reveal whether therapy selects for more differentiated or plastic states.

Proximal interactions between DMG cells and their surrounding microenvironment contribute to cellular reprogramming [30, 59]. Recent ML- and OT-based approaches, including STIFT, Moscot, and STORIES, have enabled the reconstitution of cellular trajectories while preserving spatial context in longitudinal transcriptomics datasets [23, 24, 42]. Methods such as SOCS further incorporate higher-order tissue architecture into these analyses [43]. Applied to DMG, these approaches could reveal where treatment-induced state transitions occur, identify the cellular niches that promote adaptation, and determine whether resistant cell states emerge from pre-existing populations or are induced by therapy.

## Intercellular communication networks

Treatment exposure challenges the intrinsic biology of malignant cells, but their adaptation does not occur in isolation. Tumor behavior is shaped by signals exchanged with its surrounding microenvironment, including neuron-glioma connectivity, which is increasingly recognized in the gliomagenesis of DMG [2, 19]. The TME of DMG is characterized by sparse infiltrating lymphocytes and a predominance of tumor-associated myeloid cells, actively shaped by the tumor itself [59]. Application of ligand-receptor (L-R) co-expression network inference tools, such as CellChat and CellPhoneDB, has revealed extensive DMG-myeloid communications, including the IGSF11-VISTA immune checkpoint axis (Table 1, Supplementary Table S1) [27, 44, 46, 59]. However, these methods rely on predefined populations and curated L-R databases, potentially obscuring the intra-cluster heterogeneity and interactions between uncharacterized signaling pairs [60].

Computational frameworks have therefore emerged to infer communication between individual cells, predict de novo L-R pairs, and enable multi-sample comparisons [47, 48, 60]. Using supervised contrastive learning, scComm reconstructs cell-cell communication profiles across diverse conditions, whereas CELLetter integrates large-language models, L-R co-expression, and downstream transcription factor activity to predict novel, context-specific L-R pairs [47, 60]. Tensor-based approaches, including Tensor-cell2cell, model communication across multiple biological contexts by simultaneously accounting for interactions between cellular populations, L-R pairs, and variables such as treatment or time [48]. Because paracrine and juxtacrine signaling require direct spatial proximity, OT-based methods such as COMMOT can infer communication networks between neighboring cells, enabling identification of TME interactions that may promote invasion, plasticity, or treatment response [49]. More recently, GNN-based approaches including CellNEST can reconstruct spatial communication networks by identifying L-R interactions between neighboring cells and tracing signaling pathways across multicellular niches [50]. Applied to DMG, these methods could identify TME interactions that drive cellular plasticity, immune suppression, and treatment resistance. To capture the directional nature of biological signaling, DualCellChat constructs a directed graph from spatial coordinates, inferring the role of cells as senders and receivers [51]. Characterizing these communication networks is therefore critical for understanding the signaling axes that promote treatment escape, highlighting actionable pathways that could be therapeutically disrupted to either restore antitumor immune activity or enhance treatment efficacy [27].

## Bridging the gap between computational biology and clinical care

With increasing accessibility of single-cell and spatially resolved human datasets in DMG (Supplementary Table S2), the application of emerging ML-based solutions is becoming increasingly well positioned to uncover the determinants of tumor resistance. However, translating these models and derived biomarkers into clinical practice remains a complex task, requiring extensive datasets, prospective validation, reproducibility, and ethical deployment [27].

Most computational tools require comparisons across multiple samples or timepoints, yet true longitudinal sampling is unfeasible for DMG, limited to small, heterogeneous, biopsy-limited datasets [17]. Although tissue collection is now performed routinely, tissue size, anatomical location, and quality strongly influence the model-derived outcomes [17]. Matched samples are often restricted to paired diagnostic biopsies and post-mortem autopsies, potentially confounding cumulative effects of multiple therapeutic interventions. Consequently, modeling intratumoral heterogeneity, cellular adaptation, and therapeutic resistance in real-world DMG tissues remains challenging, particularly for DL approaches, which require adequate patient-level biological replication, careful separation of training and testing datasets at the patient level to avoid information leakage, robust generalization across batches and institutions, and validation in independent cohorts. To address this gap, data collection in preclinical settings constitutes a relevant alternative, enabling tissue sampling across multiple timepoints [17, 58, 61]. Relevant models include immunocompetent, patient-derived xenografts, humanized mice, and sophisticated organoid systems [61]. Moreover, liquid biopsy (e.g., blood or cerebrospinal fluid) offers an emerging bridge for longitudinal monitoring of disease progression by non-invasive sampling. Liquid biopsy is increasingly being incorporated into DL models to classify pediatric CNS tumors, while plasma-derived biomarkers enable non-invasive monitoring of treatment response in DMG [62, 63].

Moving forward, integrating time-resolved data from preclinical models, paired patient samples and liquid biopsies could enable prospective modeling of tumor evolution, extending computational approaches beyond retrospective characterization of resistance [19, 24]. Once recurrent features of evolution are established, these models could support adaptive clinical trial designs dynamically assigning patients to biomarker-driven combination therapies before resistance emerges. Translating these models into clinical decision-making will require preclinical validation, consistency across independent clinical cohorts and prior prospective testing in clinical trials [19]. Importantly, resistance biomarkers could inform patient stratification in clinical trials by identifying individuals at high risk of treatment failure. For example, *TP53* mutation status constitutes a biomarker of poor prognosis, radioresistance [64] and reduced response to dordaviprone, whereas increased baseline expression of key TCA-related genes is associated with improved radiographic response [10, 57]. Successful clinical deployment will also require harmonized governance, data generation, preprocessing, annotation, and reporting standards across institutions to ensure model reproducibility, alongside informed patient consent and safeguards against patient-level bias and metadata privacy risks that could otherwise undermine equitable translation into patient care.

## Discussion

Therapeutic resistance remains one of the greatest challenges in oncology, yet the mechanisms underpinning treatment adaptation in DMG remain largely unknown [19]. Advances in single-cell and spatial transcriptomics technologies enable the decoding of cellular identity, function and intercellular interactions at high resolution [5, 17]. In this Review, we highlight computational-based approaches that can dissect tumor adaptation and determine whether resistance arises from selection of pre-existing cellular states, adaptive transcriptional reprogramming, cell-state transitions, or rewiring of intercellular communication networks. Applying these approaches to the growing collection of DMG datasets presents unique opportunities to understand the reasons underpinning treatment failure yet also poses significant challenges (Supplementary Table S2). The diffuse infiltrative nature of the disease and the limited availability of biopsy material constrain the collection of longitudinal patient samples. However, these limitations can be overcome in preclinical murine models, where tissue sampling and experimental conditions can be consistently controlled [58, 61]. A major unmet need is the development of computational frameworks capable of jointly modeling the multiple dimensions of tumor adaptation across multimodal datasets. This is particularly relevant in DMG, where the H3 K27 alteration and the resulting chromatin dysregulation are defining features of disease biology [3]. Integration of transcriptomic, epigenetic, proteomic and spatial data will be critical for identifying the transcription factors, chromatin regulators and regulatory networks that drive adaptive cell-state transitions, as well as the epigenetic architectures that either permit therapeutic resistance or create targetable vulnerabilities. Such approaches will be essential for distinguishing clonal selection from genuine cellular reprogramming and for uncovering the mechanistic basis of treatment response and resistance. Furthermore, foundation models pretrained on large-scale single-cell and spatial atlases, such as NicheFormer, are used for cell-type annotation and cross-cohort integration, but have not yet been applied to model therapeutic resistance trajectories specifically, but their extension to this task represents a promising future direction [65].

Importantly, the four axes of tumor adaptation described in this Review should not be considered mutually exclusive biological processes, but rather a framework for classifying computational approaches according to the aspect of tumor evolution they are designed to study. Methods such as differential abundance analysis, pseudotime inference, RNA velocity, and OT can identify shifts in cell-state composition and infer potential trajectories of cellular adaptation. However, these approaches alone cannot establish the underlying biological mechanisms driving these changes, and experimental validation remains necessary to determine whether treatment response reflects clonal selection, state transition, or a combination of both. By integrating biological insights from patient samples, preclinical models, and non-invasive monitoring approaches such as liquid biopsy, ML-based approaches have the potential to reveal evolutionary trajectories governing treatment adaptation, that could be monitored in real-time [62, 63]. Ultimately, the integration of AI-based approaches, with scientific, ethical, and operational rigor, may inform more sophisticated clinical design, evolution-informed therapeutic strategies, and synergistic combination therapies, paving the way toward a future in which DMG care is predictive, personalized, and proactive.

## Supplementary Information

Below is the link to the electronic supplementary material.

Supplementary Material 1

Supplementary Material 2

Supplementary Material 3

## Data Availability

No datasets were generated or analysed during the current study.
