# Supplementary material for: Machine learning and computational approaches to model therapeutic response and resistance in diffuse midline glioma
Source: J Neurooncol. 2026 Sep 29;179(3):104. doi: 10.1007/s11060-026-05817-8 (PMC13623780; doi:10.1007/s11060-026-05817-8)
Supplement: Supplementary file 1 [file 11060_2026_5817_MOESM1_ESM.docx]

**Supplementary Information**

**Supplementary Table S1 Legend**

Methods based primarily on statistical inference, mathematical optimization, or probabilistic modelling are classified as Statistical. OT-based methods are mathematical frameworks at the interface between statistical and ML-based approaches, as they compare probability distributions directly, without a statistical inference procedure (no hypothesis test, no likelihood) nor a trained predictive model, and will be classified in a separate “OT” category. Methods are classified as ML when they explicitly incorporate ML component involving learned representations, predictive modeling, or algorithmic training, while methods relying on neural network architectures are classified as DL, even if they use OT.

**Supplementary Table S1 Abbreviations**

Abbreviations: ANOVA = analysis of variance; CCC = cell-cell communication; DL = deep learning; FDR = false discovery rate; GAT = graph attention network; GCN = graph convolutional network; GPCCA = generalized Perron cluster cluster analysis; GRN = gene regulatory network; HMC = Hamiltonian Monte Carlo; IMC = imaging mass cytometry; k-NN = k-nearest neighbors; L-R = ligand-receptor; MERFISH = multiplexed error-robust fluorescence *in situ* hybridization; MIBI-TOF = multiplexed ion beam imaging by time of flight; ML = machine learning; MLP = multi-layer perceptron; MOFA = multi-omics factor analysis; ODE = ordinary differential equation; OT = optimal transport; PC = principal component; PCA = principal component analysis; PCSF = prize-collecting Steiner forest; seqFISH = sequential fluorescence *in situ* hybridization; scATAC-seq = single-cell assay for transposase-accessible chromatin sequencing; scRNA-seq = single-cell RNA sequencing; ST = spatial transcriptomics; STARmap = spatially-resolved transcript amplicon readout mapping; SVM = support vector machine; VAE = variational autoencoder.

**Supplementary Table S1 References**

1.	Phipson B, Sim CB, Porrello ER, Hewitt AW, Powell J, Oshlack A (2022) propeller: testing for differences in cell type proportions in single cell data. Bioinformatics 38: 4720-4726 doi:10.1093/bioinformatics/btac582

2.	Burkhardt DB, Stanley JS, 3rd, Tong A, Perdigoto AL, Gigante SA, Herold KC, Wolf G, Giraldez AJ, van Dijk D, Krishnaswamy S (2021) Quantifying the effect of experimental perturbations at single-cell resolution. Nat Biotechnol 39: 619-629 doi:10.1038/s41587-020-00803-5

3.	Zhao J, Jaffe A, Li H, Lindenbaum O, Sefik E, Jackson R, Cheng X, Flavell RA, Kluger Y (2021) Detection of differentially abundant cell subpopulations in scRNA-seq data. Proc Natl Acad Sci U S A 118: e2100293118 doi:10.1073/pnas.2100293118

4.	Büttner M, Ostner J, Müller CL, Theis FJ, Schubert B (2021) scCODA is a Bayesian model for compositional single-cell data analysis. Nature Communications 12: 6876 doi:10.1038/s41467-021-27150-6

5.	Hu G, Gold J, Chitra U, Joshi S, Raphael BJ (2025) Anomaly detection in spatial transcriptomics via spatially localized density comparison. Bioinformatics 41: i493-i501 doi:10.1093/bioinformatics/btaf242

6.	Ali M, Kuijs M, Hediyeh-Zadeh S, Treis T, Hrovatin K, Palla G, Schaar AC, Theis FJ (2024) GraphCompass: spatial metrics for differential analyses of cell organization across conditions. Bioinformatics 40: i548-i557 doi:10.1093/bioinformatics/btae242

7.	Ji Z, Ji H (2016) TSCAN: Pseudo-time reconstruction and evaluation in single-cell RNA-seq analysis. Nucleic Acids Res 44: e117 doi:10.1093/nar/gkw430

8.	Wolf FA, Hamey FK, Plass M, Solana J, Dahlin JS, Gottgens B, Rajewsky N, Simon L, Theis FJ (2019) PAGA: graph abstraction reconciles clustering with trajectory inference through a topology preserving map of single cells. Genome Biol 20: 59 doi:10.1186/s13059-019-1663-x

9.	Gupta R, Cerletti D, Gut G, Oxenius A, Claassen M (2022) Simulation-based inference of differentiation trajectories from RNA velocity fields. Cell Reports Methods 2 doi:10.1016/j.crmeth.2022.100359

10.	Gayoso A, Weiler P, Lotfollahi M, Klein D, Hong J, Streets A, Theis FJ, Yosef N (2024) Deep generative modeling of transcriptional dynamics for RNA velocity analysis in single cells. Nat Methods 21: 50-59 doi:10.1038/s41592-023-01994-w

11.	Cui H, Maan H, Vladoiu MC, Zhang J, Taylor MD, Wang B (2024) DeepVelo: deep learning extends RNA velocity to multi-lineage systems with cell-specific kinetics. Genome Biol 25: 27 doi:10.1186/s13059-023-03148-9

12.	Lange M, Bergen V, Klein M, Setty M, Reuter B, Bakhti M, Lickert H, Ansari M, Schniering J, Schiller HB, Pe'er D, Theis FJ (2022) CellRank for directed single-cell fate mapping. Nat Methods 19: 159-170 doi:10.1038/s41592-021-01346-6

13.	Sha Y, Qiu Y, Zhou P, Nie Q (2024) Reconstructing growth and dynamic trajectories from single-cell transcriptomics data. Nat Mach Intell 6: 25-39 doi:10.1038/s42256-023-00763-w

14.	Huguet G, Magruder DS, Tong A, Fasina O, Kuchroo M, Wolf G, Krishnaswamy S (2022) Manifold Interpolating Optimal-Transport Flows for Trajectory Inference. Adv Neural Inf Process Syst 35: 29705-29718

15.	Li G, Kim HJ, Pendyala S, Zhang R, Vert JP, Disteche CM, Deng X, Fowler DM, Noble WS (2025) Sceptic: pseudotime analysis for time-series single-cell sequencing and imaging data. Genome Biol 26: 209 doi:10.1186/s13059-025-03679-3

16.	Haghverdi L, Büttner M, Wolf FA, Buettner F, Theis FJ (2016) Diffusion pseudotime robustly reconstructs lineage branching. Nature Methods 13: 845-848 doi:10.1038/nmeth.3971

17.	Shen X, Zuo L, Ye Z, Yuan Z, Huang K, Li Z, Yu Q, Zou X, Wei X, Xu P, Deng Y, Jin X, Xu X, Wu L, Zhu H, Qin P (2025) Inferring cell trajectories of spatial transcriptomics via optimal transport analysis. Cell Syst 16: 101194 doi:10.1016/j.cels.2025.101194

18.	Browaeys R, Saelens W, Saeys Y (2020) NicheNet: modeling intercellular communication by linking ligands to target genes. Nature Methods 17: 159-162 doi:10.1038/s41592-019-0667-5

19.	Hu Y, Peng T, Gao L, Tan K (2021) CytoTalk: De novo construction of signal transduction networks using single-cell transcriptomic data. Sci Adv 7: eabf1356 doi:10.1126/sciadv.abf1356

20.	Jin Z, Tang Z, Li X, Zhang K, Xie Z, Zhang N (2026) scComm: a contrastive learning framework for deciphering cell-cell communications at single-cell resolution. Genome Biol 27 doi:10.1186/s13059-026-04043-9

21.	Raredon MSB, Yang J, Kothapalli N, Lewis W, Kaminski N, Niklason LE, Kluger Y (2023) Comprehensive visualization of cell-cell interactions in single-cell and spatial transcriptomics with NICHES. Bioinformatics 39 doi:10.1093/bioinformatics/btac775

22.	Li Z, Wang T, Liu P, Huang Y (2023) SpatialDM for rapid identification of spatially co-expressed ligand-receptor and revealing cell-cell communication patterns. Nat Commun 14: 3995 doi:10.1038/s41467-023-39608-w

23.	Shao X, Li C, Yang H, Lu X, Liao J, Qian J, Wang K, Cheng J, Yang P, Chen H, Xu X, Fan X (2022) Knowledge-graph-based cell-cell communication inference for spatially resolved transcriptomic data with SpaTalk. Nat Commun 13: 4429 doi:10.1038/s41467-022-32111-8

24.	Cang Z, Nie Q (2020) Inferring spatial and signaling relationships between cells from single cell transcriptomic data. Nat Commun 11: 2084 doi:10.1038/s41467-020-15968-5

25.	Yuan Y, Bar-Joseph Z (2020) GCNG: graph convolutional networks for inferring gene interaction from spatial transcriptomics data. Genome Biol 21: 300 doi:10.1186/s13059-020-02214-w

26.	Yang W, Wang P, Xu S, Wang T, Luo M, Cai Y, Xu C, Xue G, Que J, Ding Q, Jin X, Yang Y, Pang F, Pang B, Lin Y, Nie H, Xu Z, Ji Y, Jiang Q (2024) Deciphering cell-cell communication at single-cell resolution for spatial transcriptomics with subgraph-based graph attention network. Nat Commun 15: 7101 doi:10.1038/s41467-024-51329-2

27.	Dimitrov D, Schafer PSL, Farr E, Rodriguez-Mier P, Lobentanzer S, Badia IMP, Dugourd A, Tanevski J, Ramirez Flores RO, Saez-Rodriguez J (2024) LIANA+ provides an all-in-one framework for cell-cell communication inference. Nat Cell Biol 26: 1613-1622 doi:10.1038/s41556-024-01469-w

28.	Tanevski J, Flores ROR, Gabor A, Schapiro D, Saez-Rodriguez J (2022) Explainable multiview framework for dissecting spatial relationships from highly multiplexed data. Genome Biol 23: 97 doi:10.1186/s13059-022-02663-5

29.	Yang Y, Li G, Zhong Y, Xu Q, Lin YT, Roman-Vicharra C, Chapkin RS, Cai JJ (2023) scTenifoldXct: A semi-supervised method for predicting cell-cell interactions and mapping cellular communication graphs. Cell Syst 14: 302-311 e304 doi:10.1016/j.cels.2023.01.004

**Supplementary Table S2 Abbreviations**

Abbreviations: scRNAseq = single-cell RNA sequencing; snRNAseq = single-nucleus RNA sequencing; ST = spatial transcriptomics.

**Supplementary Table S2 References**

1.	Jessa S, Mohammadnia A, Harutyunyan AS, Hulswit M, Varadharajan S, Lakkis H, Kabir N, Bashardanesh Z, Hébert S, Faury D, Vladoiu MC, Worme S, Coutelier M, Krug B, Faria Andrade A, Pathania M, Bajic A, Weil AG, Ellezam B, Atkinson J, Dudley RWR, Farmer J-P, Perreault S, Garcia BA, Larouche V, Blanchette M, Garzia L, Bhaduri A, Ligon KL, Bandopadhayay P, Taylor MD, Mack SC, Jabado N, Kleinman CL (2022) K27M in canonical and noncanonical H3 variants occurs in distinct oligodendroglial cell lineages in brain midline gliomas. Nature Genetics 54: 1865-1880 doi:10.1038/s41588-022-01205-w

2.	Liu I, Jiang L, Samuelsson ER, Marco Salas S, Beck A, Hack OA, Jeong D, Shaw ML, Englinger B, LaBelle J, Mire HM, Madlener S, Mayr L, Quezada MA, Trissal M, Panditharatna E, Ernst KJ, Vogelzang J, Gatesman TA, Halbert ME, Palova H, Pokorna P, Sterba J, Slaby O, Geyeregger R, Diaz A, Findlay IJ, Dun MD, Resnick A, Suva ML, Jones DTW, Agnihotri S, Svedlund J, Koschmann C, Haberler C, Czech T, Slavc I, Cotter JA, Ligon KL, Alexandrescu S, Yung WKA, Arrillaga-Romany I, Gojo J, Monje M, Nilsson M, Filbin MG (2022) The landscape of tumor cell states and spatial organization in H3-K27M mutant diffuse midline glioma across age and location. Nat Genet 54: 1881-1894 doi:10.1038/s41588-022-01236-3

3.	Ren Y, Huang Z, Zhou L, Xiao P, Song J, He P, Xie C, Zhou R, Li M, Dong X, Mao Q, You C, Xu J, Liu Y, Lan Z, Zhang T, Gan Q, Yang Y, Chen T, Huang B, Yang X, Xiao A, Ou Y, Su Z, Chen L, Zhang Y, Ju Y, Zhang Y, Wang Y (2023) Spatial transcriptomics reveals niche-specific enrichment and vulnerabilities of radial glial stem-like cells in malignant gliomas. Nat Commun 14: 1028 doi:10.1038/s41467-023-36707-6

4.	DeSisto J, Donson AM, Griesinger AM, Fu R, Riemondy K, Mulcahy Levy J, Siegenthaler JA, Foreman NK, Vibhakar R, Green AL (2024) Tumor and immune cell types interact to produce heterogeneous phenotypes of pediatric high-grade glioma. Neuro Oncol 26: 538-552 doi:10.1093/neuonc/noad207

5.	LaBelle JJ, Haase RD, Beck A, Haase J, Jiang L, Oliveira de Biagi CA, Jr., Neyazi S, Englinger B, Liu I, Trissal M, Jeong D, Hack OA, Nascimento A, Shaw ML, Nguyen CM, Castellani S, Mathewson ND, Ashenberg O, Veiga Cruzeiro GA, Rosenberg T, Vogelzang JR, Pyrdol J, Marx S, Luomo AM, Godicelj A, Baumgartner A, Rozowsky JS, Madlener S, Mayr L, Peyrl A, Geyeregger R, Loetsch D, Dorfer C, Haberler C, Stepien N, Slavc I, Davidson TB, Prins RM, Yeo KK, Cooney T, Ligon K, Lidov H, Alexandrescu S, Baird LC, Gojo J, Wucherpfennig KW, Filbin MG (2025) Dissecting the immune landscape in pediatric high-grade glioma reveals cell state changes under therapeutic pressure. Cell Rep Med 6: 102095 doi:10.1016/j.xcrm.2025.102095

6.	van den Broek TJM, Hoogendijk R, Kranendonk MEG, Lammers JAS, Krishnamoorthy AL, van Ineveld RL, Molleson M, Tsvetkov VO, Ringnalda FCA, van de Wetering M, Su Y, Bianco JI, Ruiz-Moreno C, Ries MG, Hoving EW, van der Lugt J, Akkari L, Schrijver DP, Stunnenberg HG, Rios AC, van Vuurden DG, Zomer A (2025) Single-cell spatial analysis of pediatric high-grade glioma reveals a novel population of SPP1+/GPNMB+ myeloid cells with immunosuppressive and tumor-promoting capabilities. Neuro-Oncology Pediatrics 1 doi:10.1093/neuped/wuaf002

7.	Kordowski A, Mulay O, Tan X, Vo T, Baumgartner U, Maybury MK, Hassall T, Harris L, Nguyen Q, Day BW (2025) Spatial analysis of a complete DIPG-infiltrated brainstem reveals novel ligand-receptor mediators of tumour-to-TME crosstalk. Acta Neuropathol Commun 13: 35 doi:10.1186/s40478-025-01952-x

8.	Webb MG, Chow F, McCullough CG, Zhang B, Lee JJY, Bassiouni R, Garrett NE, Hurth K, Carpten JD, Zada G, Craig DW (2025) Resolving spatial subclonal genomic heterogeneity of loss of heterozygosity and extrachromosomal DNA in gliomas. Nature Communications 16: 5290 doi:10.1038/s41467-025-59805-z

9.	Sussman JH, Oldridge DA, Yu W, Chen CH, Zellmer AM, Rong J, Parvaresh-Rizi A, Thadi A, Yang A, Tumulty JS, Xiong B, Wu DW, Sun Y, Bandyopadhyay S, Xu J, Brosius S, Barkardottir UY, Seka I, Ahn KJ, Elghawy O, Baxter AE, Koptyra MP, Vanguri RS, McGrory S, Storm PB, Amankulor NM, Santi M, Viaene AN, Zhang N, De Raedt T, Cole K, Tan K (2026) A longitudinal single-cell and spatial multiomic atlas of pediatric high-grade glioma. Cell Rep Med 7: 102766 doi:10.1016/j.xcrm.2026.102766

10.	Collot R, Ruiz-Moreno C, Honhoff C, van den Broek TJM, Wezenaar AKL, Kloosterman DJ, Ariese HCR, Johnson H, Vervoort BMT, Jeiroshi A, Bunt J, van Ineveld RL, Bokobza E, Rebel HG, Te Pas BM, Ringnalda FCA, van de Wetering M, Robe PA, Kool M, Cochran JR, Kranendonk MEG, van Vuurden DG, Hulleman E, Wehrens EJ, Zomer A, Stunnenberg HG, Rios AC (2026) IGSF11-VISTA is a critical and targetable immune checkpoint axis in diffuse midline glioma. Cancer Cell 44: 641-657 e649 doi:10.1016/j.ccell.2025.12.020

11.	Okada M, Subramaniam B, Geng B, Jung J, Yu B, Lin F, Fu Y, Wang Y, Yu W, Laternser S, Gupta N, Müller S, Wang L, Diaz A, Nazarian J (2026) Single-nucleus profiling of postmortem diffuse midline gliomas identifies mitochondrial biogenesis as a resistance mechanism to imipridone therapy. Neuro Oncol doi:10.1093/neuonc/noag119
